# The Role of the CoREST/REST Repressor Complex in Herpes Simplex Virus 1 Productive Infection and in Latency

**DOI:** 10.3390/v5051208

**Published:** 2013-04-29

**Authors:** Guoying Zhou, Te Du, Bernard Roizman

**Affiliations:** Marjorie B. Kovler Viral Oncology Laboratories, The University of Chicago, 910 East 58th Street, Chicago, IL 60637, USA

**Keywords:** HCLR, herpes viruses, productive infection, latency

## Abstract

REST is a key component of the HDAC1 or 2, CoREST, LSD1, REST (HCLR) repressor complex. The primary function of the HCLR complex is to silence neuronal genes in non-neuronal cells. HCLR plays a role in regulating the expression of viral genes in productive infections as a donor of LDS1 for expression of α genes and as a repressor of genes expressed later in infection. In sensory neurons the HCLR complex is involved in the silencing of viral genome in the course of establishment of latency. The thesis of this article is that (a) sensory neurons evolved a mechanism to respond to the presence and suppress the transmission of infectious agents from the periphery to the CNS and (b) HSV evolved subservience to the HCLR with at least two objectives: to maintain a level of replication consistent with maximal person-to-person spread and to enable it to take advantage of neuronal innate immune responses to survive and be available for reactivation shielded from adaptive immune responses of the host.

## 1. Background

Herpes simplex viruses 1 and 2 (HSV-1 and HSV-2) replicate and destroy cells at the portal of entry into the body (mouth, genitals), but remain latent without obvious effects in sensory ganglia. The same viruses readily replicate and destroy neurons maintained in culture [1,2]. The fundamental question posed in numerous studies over the past century is how a virus that efficiently and vigorously replicates and can cause massive tissue destruction is nevertheless silenced in sensory ganglia. In the absence of any data, two hypotheses could explain the biology of HSV. The first is that HSV evolved two sets of regulatory pathways, one for viral gene expression at the portal of entry into the body, and a totally different pathway for viral gene expression in sensory ganglia. The alternative hypothesis is based in part on the observation that HSV recruits host proteins to perform the functions it needs. In this context, the host proteins available for recruitment in cells at the portal of entry into the body and in sensory neurons could be different. 

## 2. Productive Infection

The mantra of HSV-1 gene expression modified in recent years is that HSV genes form several groups that are coordinately and sequentially derepressed [1]. Thus, on entry into the nucleus HSV-1 DNA is immediately bound by repressive histones and cellular repressors. The α genes are derepressed first, followed by the β and γ genes [2]. The focus of the studies carried out at the time of the discovery of the role of the CoREST/REST repressor complex was on ICP0, an HSV multifunctional regulatory protein [3,4,5]. Thus, at low multiplicity of infection with ∆ICP0 mutants α genes are expressed, but the transition from α to β gene expression does not ensue [2,6,7]. One “desperate” hypothesis we pursued is that ICP0 mimics a less efficient cellular protein since, at high multiplicity of infection ∆ICP0 mutants do replicate. To our surprise, a blast analysis of human genes known at that time revealed that a stretch of approximately 70 ICP0 residues at the amino terminus of CoREST were conserved with relatively good homology. We demonstrated that (a) CoREST and ICP0 interacts, (b) the binding site of ICP0 in CoREST corresponded to the homologous sequence in the amino terminal of the protein. The binding site for CoREST in ICP0 was immediately downstream of the homologous sequence [4,8].

CoREST and REST are components of a larger complex, consisting minimally of histone deacetylase (HDAC)/corepressor element-1 silencing transcription factor (CoREST)/lysine specific demethylase1(LSD1)/RE1-silencing transcription factor (REST) repressor complex (HCLR) [9,10,11,12,13]. In this complex CoREST binds HDACs, LSD1 and REST [9]. The primary function of this complex is to repress neuronal genes in non-neuronal cells [14,15,16,17,18]. REST is usually absent or in very low amounts in neurons [19,20,21,22]. Stressed neurons, for example in Huntington’s disease, may express REST and this may lead to their death [23,24,25,26]. REST binds to a somewhat degenerate response element known as RE1 [27,28,29,30]. Analyses of the HSV DNA sequence predicts numerous REST binding sites scattered through the genome (unpublished data).

**Figure 1:**
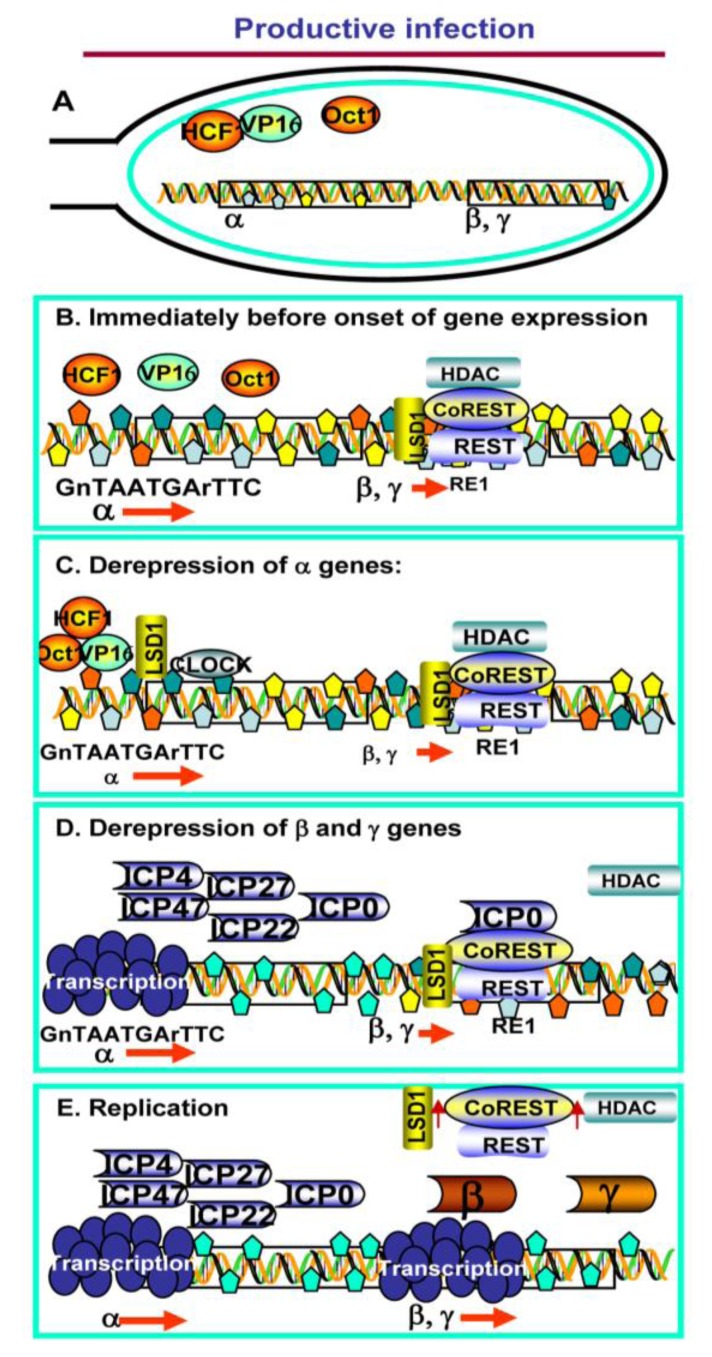
Schematic representation of the role of the HCLR complex in productive infection. (**A**) HSV DNA entering the nucleus is bereft of bound proteins. Several viral proteins are introduced into the cell, and ultimately into the nucleus, during infection; among these VP16 is shown here; (**B**) Within a very short time frame viral DNA is coated by repressive histones and interacts with the HCLR complex. In addition (not shown here) ND10 nuclear bodies assemble at the viral DNA; (**C**) VP16 recruits HCF1, Oct1, LSD1 and other transcriptional factors to derepress and transcribe α genes. Concurrently (not shown), the ND10 bodies disassemble; (**D**) ICP0, one of the α proteins, binds to CoREST and dislodges HDAC1. Transcription of β and γ genes ensues; (**E**) Late in infection, after the onset of viral DNA synthesis, a fraction of LSD1, CoREST, REST and HDAC 1 is translocated to the cytoplasm.

To assess the significance of the interaction between ICP0 and CoREST, three series of experiments were done. In the first the open reading frames expressing ICP0 were replaced with those encoding a dominant negative CoREST lacking the binding sites of ICP0 and HDAC1. The mutant replicated 10 to 100 fold better than ∆ICP0 mutant, indicating that a key function of ICP0 was to block the repressive action of HCLR complex [8]. In the second series of experiments the binding site of CoREST on ICP0 was mutagenized by codon substitution. In wild type infected cells ICP0 colocalizes first with ND10 bodies in the nucleus [31,32]. Within a few hours, however, key components of ND10 are degraded, the constituents of ND10 are dispersed, and ICP0 becomes dispersed in the nucleus. Between 6 and 9 h after infection ICP0 is fully localized in the cytoplasm [33,34]. ICP0 mutants lacking the CoREST binding site were largely retained in the nucleus [4]. Finally, the conclusion that ICP0 blocks the repressive functions of the HLCR complex was supported by the evidence that ICP0 displaced HDAC1 from the complex [5,8]. Late in infection at least a fraction of HDAC1, CoREST, LSD1 and REST were exported into the cytoplasm. The translocation of the HCLR components was delayed in the presence of inhibitors of viral DNA synthesis [3]. A schematic representation of this model is shown in Figure 1.

The studies described above indicate that HCLR complex was a key component in repressing the transition from β to γ gene expression. Curiously, this complex, at least indirectly, plays a role in the initiation of expression of α gene. Thus, to initiate gene expression, VP16, a virion protein introduced into the cell during infection, recruits several proteins to the promoters of α genes, especially Host Cell Factor 1 (HCF1), Octamer binding protein 1(Oct1) and LSD1 [1,2]. LSD1 is unstable in the absence CoREST [17,35,36]. Indeed, silencing of either CoREST or REST by siRNA decreased the levels of LSD1 and reduced the expression of α genes [37]. 

The fundamental conclusion of these studies is that on entry of viral DNA into the nucleus all viral genes are repressed and that the HCLR complex plays a key role in the expression of viral genes during productive infection. The obvious question is why it plays this role. Viruses can evolve faster than the cells they infect. In the course of its evolution, HSV could have readily escaped from the repressive effects of the HCLR complex through base substitution. Based on the effectiveness of the repressive effects of the HCLR complex, it would appear that HSV evolved to embrace the HCLR repressor rather than avoid it. In evolving ICP0, HSV has effectively nullified the repressive effects of the HCLR complex. One hypothesis to explain the interaction with HCLR is that HSV needs it to establish a latent state.

## 3. Establishment of Latency and Reactivation

REST is not a normal constituent of neurons [19,20,21,22]. To play a role in the establishment of latency, REST would have to be induced or recruited from satellite cells. As reported elsewhere, REST was detected in immunoblots of extracts from whole murine trigeminal ganglia, but not in extracts of an intact mouse brain [38]. One way to test whether it plays a role in establishment of latency is to insert a dominant negative (dn) REST, driven by an SV40 promoter, into the HSV genome. The dnREST we constructed contains the DNA binding domain, but lacks the N and C terminal domains required for binding the repressive components of the complex [38]. The prediction would be that if the HCLR complex plays no role in the establishment of latency, the expression of a dominant negative REST would have no effect. If REST is induced or recruited by the entry of the virus into the neuron, the dominant negative REST would be predicted to compete with wild type REST, bind to viral DNA and preclude its silencing. In all, 3 viruses were constructed, *i.e.*, a virus encoding a dnREST, a virus encoding a wild-type rest and finally, a virus containing a series of stop codons at the site of insertion of the REST genes [38]. In these experiments, mice were inoculated by the corneal route. The trigeminal ganglia were harvested at frequent intervals during the first two weeks and at the end of the test period (28–30 days). The key findings were that the virus carrying the gene encoding the dnREST was more virulent and replicated to higher titers than either the wild type virus or the control viruses, *i.e.*, those encoding wild-type rest or carrying the string of stop codons. Specifically, we observed higher levels of HSV in mouse brains, total destruction of trigeminal ganglia, higher amounts of virus in the brain and high mortality even when the size of the inoculum was reduced. The enhanced virulence of the virus encoding dnREST was verified by inoculating mice by the intraperitoneal route [39]. 

The studies on the dnREST led to 2 conclusions. As construed by the experimental design, higher virulence suggests that dnREST competed with wild-type REST and blocked the HCLR complex. Confirmatory results emerged as described below in the section on virus reactivation.

Perhaps the most significant and unexpected finding to emerge from these experiments is that the dnREST virus was more virulent than the wild-type parent [1]. Implicit in this finding is the proposition that HSV could have evolved to be far more virulent than it is. A necessary extension of this proposition is that, in the course of its evolution, HSV was constrained from replicating in every cell it infects and from efficient spread from the site of infection to target organs (e.g., brain, liver, *etc.*) that could cause the infected individual to succumb to infection. Also implicit in this proposition is that the HCLR complex plays a role in defining the virulence of the virus, in that it blocks viral replication in a significant fraction of the cells it infect *in* *vivo*. The failure to evolve into a more pathogenic virus is not unexpected: highly virulent viruses that kill their hosts rapidly are defective in spreading. Another way to put it is that very sick people do not efficiently spread a sexually transmitted virus. 

## 4. Reactivation from Latency

HSV reactivates in response to environmental, hormonal and emotional stress [39,40,41]. On reactivation, HSV multiplies and is translocated to a site at or near the portal of entry [39,40]. Reactivation presents two problems. The first is how diverse stimuli, that at times are highly individual, cause latent virus to reactivate. The second puzzle is the mechanism of reactivation. In latently infected neurons, viral DNA forms an episome [2,42,43]. As noted earlier, the only viral gene products accumulating in neurons are a long, non-coding RNA, designated the Latency Associated Transcript (LAT) [44,45,46,47,48,49,50], and a set of micro RNAs (miRNAs) [51,52,53,54]. 

Viral DNA itself is in the form of facultative heterochromatin [42,55,56,57,58]. The preeminent question is how HSV reactivates from the silent, repressed state in the absence of VP16 and ICP0. The question also arises as to the role, if any, of the CoREST/REST complex in the reactivation from latent state.

The model we have chosen to resolve these questions are organ cultures of trigeminal ganglia harvested 30 days after corneal inoculation [59]. Incubation of ganglia in medium containing the antibody to NGF causes the virus to reactivate. Incubation in medium containing NGF and EGF delays reactivation [59]. The relevant finding obtained to date is that in medium containing anti-NGF antibody, viral mRNAs representative of all classes of viral genes exhibit an increase in amounts, beginning 5 h after excision and incubation of the ganglia. Concurrently the LAT and miRNAs decrease in amount [59]. Reactivation of all genes also takes place in the presence of cycloheximide, indicating that (a) activation does not require prior protein synthesis, and (b) the reactivation in this system is a catastrophic event that causes total derepression of the viral genome. Finally, in this system a wild-type virus carrying wild-type REST does not reactivate. Reactivation, as measured by transcription of all viral genes, does occur if the ganglia infected with the virus encoding REST are incubated in medium containing cycloheximide, either for the entire 24 h interval or for at least 5 hr interval after excision [60]. 

**Figure 2:**
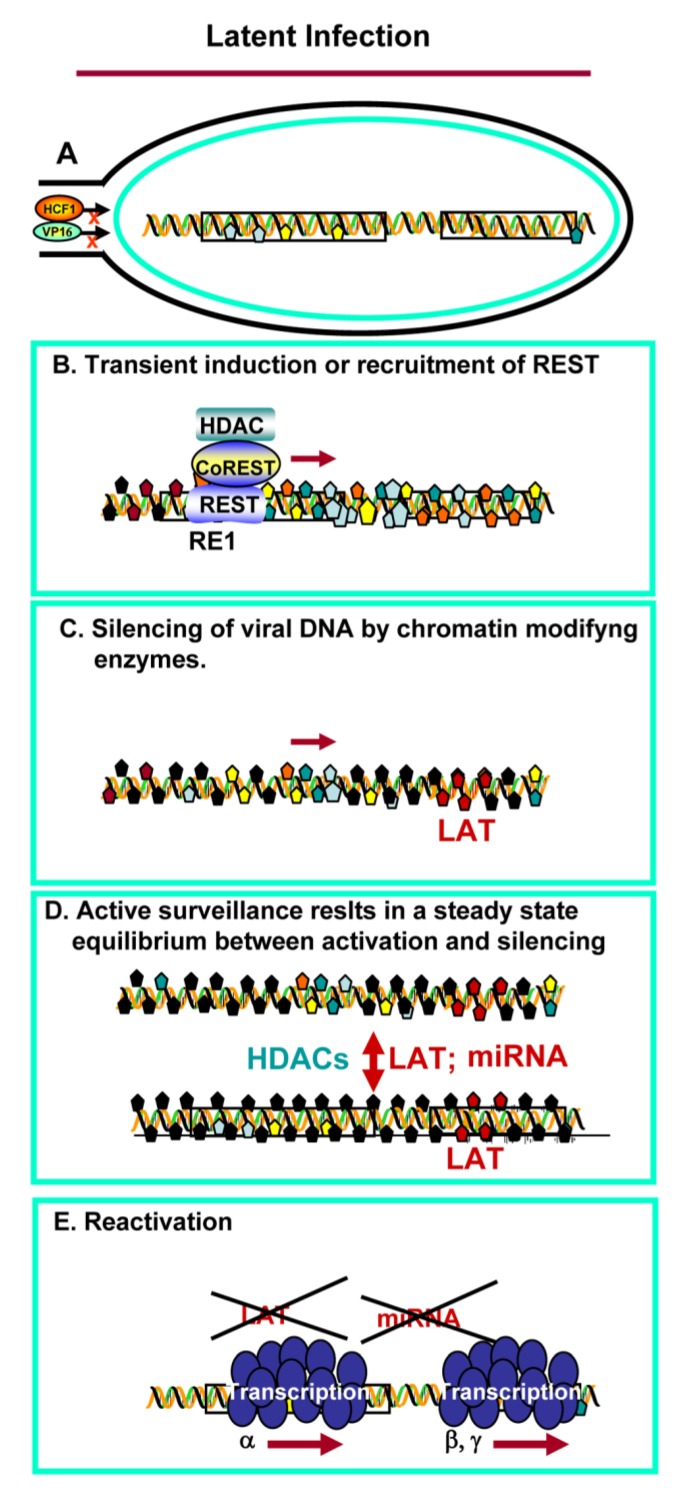
Schematic representation of the role of the HCLR complex in HSV latency. (**A**) Viral DNA is released into the neuronal nucleus free of bound DNA. Available data suggest that HCF1 and VP16 do enter the nucleus; (**B**) REST is induced or recruited from satellite cells. The HCLR repressor initiates silencing of the viral genome; (**C**) HSV DNA protein complex is modified by chromatin modifying enzymes; (**D**) HSV is in a latent (silent) state. The neuron is surveyed by LAT, miRNAs. The state of repression requires the presence of active HDACs except for transcription of the LAT and miRNAs; (**E**) On reactivation all viral genes are expressed at once. The LAT and miRNAs are degraded. There is no evidence that REST is induced or recruited during reactivation.

The conclusion drawn from these studies is that the CoREST/REST complex plays a role in the establishment of latency, but not in reactivation. A model of the establishment of latency is shown schematically in Figure 2. 

## 5. Conclusions: The Role of the CoREST/REST Repressor Complex in the HSV Lifestyle

The fundamental findings summarized above indicate that the CoREST/REST complex is involved in the suppression of viral gene expression, in both productive infection and in the establishment of latent, silent infections in sensory neurons. As noted above, HSV could have readily escaped interactions with the CoREST/REST repressor complex, by base substitution. One interpretation of the data is that in fact HSV subserved itself to the repressor complex. The data posit the following:

(i) All of the data available to date indicate that no viral function is required to establish latent infections [2,42]. Silencing of viral DNA in neurons appears to be a neuronal function, most likely a defense mechanism to block transmission of viruses from the periphery to the central nervous system. Viewed from this prospective, HSV took advantage of the innate neuron defenses to enable itself to be silenced and remain as a reservoir in its human host.
(ii) One response to stress in neurons is activation of the CoREST/REST complex. This has been reported in some degenerative diseases of the CNS [23,24,25,26]. It is conceivable that transient expression of REST following entry of the virus into the CNS, is sufficient to initiate the epigenetic modifications essential to silence viral DNA, but not irreversibly damage the neuron harboring the virus in the silent state.
(iii) In cultured cells productively infected with HSV-1, the CoREST/REST repressor complex is readily overcome by displacement of HDACs from the repressor complex by ICP0, or in high multiplicity infections [8]. The studies on the HSV-1 mutant carrying dnREST [38] suggest that the interaction with the CoREST/REST repressor complex serves to maintain equilibrium between excessive replication—that would irreversibly injure the host and prevent transmission—and minimal replication—which is insufficient to secure establishment of latent virus, and ultimately frequent transmission from infected to uninfected individuals.

Viewed in this light, the hypothesis driving these studies is that HSV entering sensory neurons triggers transient synthesis or recruitment of REST from satellite cells. The transiently assembled repressor complex initiates the silencing of viral DNA by cellular enzymes. The observation that the virus encoding wild-type REST does not reactivate, suggests that REST is not activated in neurons harboring virus and deprived of NGF [60]. Key questions—which mechanisms suppresses the continued presence of REST in neurons harboring latent virus, or mechanisms that lead to the massive derepression of viral genome during reactivation—remain unanswered.
